# Mental Health and Drivers of Need in Emergent and Non-Emergent Emergency Department (ED) Use: Do Living Location and Non-Emergent Care Sources Matter?

**DOI:** 10.3390/ijerph15010129

**Published:** 2018-01-13

**Authors:** Moira C. McManus, Robert J. Cramer, Maureen Boshier, Muge Akpinar-Elci, Bonnie Van Lunen

**Affiliations:** 1 Community and Environmental Health, College of Health Sciences, Old Dominion University; Norfolk, VA 757-683-4259, USA; mcros014@odu.edu (M.C.M.); mboshier@odu.edu (M.B.); makpinar@odu.edu (M.A.-E.); 2 Physical Therapy and Athletic Training, College of Health Sciences, Old Dominion University; Norfolk, VA 757-683-4519, USA; bvanlune@odu.edu

**Keywords:** emergency department, mental health, non-emergent care, Health Services Use, living location

## Abstract

Emergency department (ED) utilization has increased due to factors such as admissions for mental health conditions, including suicide and self-harm. We investigate direct and moderating influences on non-emergent ED utilization through the Behavioral Model of Health Services Use. Through logistic regression, we examined correlates of ED use via 2014 New York State Department of Health Statewide Planning and Research Cooperative System outpatient data. Consistent with the primary hypothesis, mental health admissions were associated with emergent use across models, with only a slight decrease in effect size in rural living locations. Concerning moderating effects, Spanish/Hispanic origin was associated with increased likelihood for emergent ED use in the rural living location model, and non-emergent ED use for the no non-emergent source model. ‘Other’ ethnic origin increased the likelihood of emergent ED use for rural living location and no non-emergent source models. The findings reveal ‘need’, including mental health admissions, as the largest driver for ED use. This may be due to mental healthcare access, or patients with mental health emergencies being transported via first responders to the ED, as in the case of suicide, self-harm, manic episodes or psychotic episodes. Further educating ED staff on this patient population through gatekeeper training may ensure patients receive the best treatment and aid in driving access to mental healthcare delivery changes.

## 1. Introduction

### 1.1. Background

Non-emergent use of the emergency department (ED) is a growing national concern, accounting for a usage rate of 42.8 visits per 100 people per year [1]. The use of EDs for non-emergent care results in overcrowding, strains limited resources [2,3], and can result in fragmented care [4,5]. Current use of EDs is shaped by the view of many people as an around-the-clock healthcare resource that is available for patients to use for healthcare needs, emergent or not [6]. Non-emergent ED use resulted in a doubling of the total United States ED expenditure between 2000 and 2008 [7] and is considered by the medical community to be ‘excess charges’ [8].

Previous research has found various reasons for non-emergent ED use including unavailability of medical resources, primary-care access, insurance status, demographic factors, and chronic disease/illness status. Seeking repeated non-emergent ED treatment has been shown to be due to living in an area lacking in medical resources [3,9,10,11,12,13,14]. Not having access to primary-care providers may be the result of needing to be seen after normal primary-care office hours or on weekends [14,15,16]. In other instances, provider practices may not be taking new patients [17,18]. Many primary-care providers do not take Medicare, Medicaid or other public insurance patients, do not accept patients who are uninsured, or have a patient population that consists of the maximum number of these patients that they can manage [3,19,20,21,22]. Characteristics such as age, race and sex have also been shown to influence non-emergent ED use [7,21,23].

### 1.2. Drivers of Emergency Department (ED) Use: Impact of Chronic Disease and Mental Illness on Non-Emergent ED Use

Non-emergent ED use has also been shown to be due to patients’ lack of knowledge in managing a previously diagnosed mental or chronic illness [10,13,24,25], such as anxiety disorders [21], personality disorders [26], obesity [27], diabetes [13], asthma [10,23,24,25], and pain diagnoses [26]. Doran and colleagues [26] showed that there are typically four factors associated with high ED use: schizophrenia, homelessness, opioid prescription use, and heart failure. This is consistent with the factors that were found to be associated with ED use in a study by Pines [22] which reported that high users are typically of low socioeconomic status (SES) and have a chronic and/or a mental illness. Additionally, individuals with chronic disease or illness, including mental health conditions and substance-abuse disorders, may have a higher requirement for care since they typically need more monitoring [28]. For example, Schizophrenia has previously been associated with high ED use [22,26]. Without access to a primary-care provider, an individual with a mental or chronic medical condition is more likely to seek non-emergent ED care [22]. Interestingly, the literature generally lacks a thorough examination of whether mental illness-driven ED use is differentially associated with emergent versus non-emergent use; this fact is particularly critical for ED providers, because many mental health concerns (e.g., substance use, suicide) may by definition be emergent, and yet other conditions (e.g., schizophrenia, anxiety) may be non-emergent. ED staff would benefit from workplace education informing the level of emergent-care usage associated with mental health concerns.

### 1.3. Potential Moderators: Living Location and Non-Emergent Sources

Rural living locations account for approximately 20% of the U.S. population [29], yet less than 10% of primary-care providers practice in these areas [9]. This has been shown to be influential in non-emergent ED use in rural locations, as 50% of ED visits made by individuals living in a rural area have been non-emergent [9]. The number of non-emergent care sources in a location has also been shown to be influential in one’s use of the ED for non-emergent care [30]. An individual will seek out care based on what is available to them [31]. ED use drastically increases when the number of outpatient providers in a location decreases [32]. Therefore, EDs are often picked when another source of non-emergent care is not available [3,31]. These geography-dependent factors may moderate the impact of drivers of non-emergent ED usage, yet such a nuanced examination of non-emergent ED use is currently lacking in the literature. We address this using an established conceptual framework in the health services research literature.

### 1.4. The Behavioral Model of Health Services Use

The original Aday-Andersen Behavioral Model of Health Services Use consists of three constructs [33]. Predisposing characteristics influence one’s predisposition to use a healthcare resource [33]. Enabling resources are personal, family, and community influences on one’s decision to use healthcare resources [33]. Need examines one’s health and functional status, both perceived and evaluated, for their effects on the use of healthcare resources. Perceived need is considered subjective, and evaluated need is objective [34] and typically measured through either professional judgment or professional diagnosis [33]. The onset or maintenance of a chronic medical illness or the presence of mental-illness concerns fit within a number of typical need variables that may drive non-emergent health service utilization.

### 1.5. Present Study

Several studies have examined predictors of ED use, such as mental health (see above review), but the potential moderating role of sociological or contextual influences such as living location and availability of non-emergent care sources remains unexplored. This study examined the influence of predisposing factors (i.e., age, sex, race, and ethnicity), enabling factors (i.e., access to health insurance, day of the week of visit, time of day of visit, living location, and non-emergent care sources), and need variables (i.e., chronic disease admissions, any presence of a chronic disease, and mental health admissions) on ED use. The hypotheses for this study are:

**Hypothesis** **1** **(H1).** It is hypothesized that need variables will exert the largest effects on ED usage. Specifically, chronic disease or illness admissions will, compared to other admissions, be associated with an increased likelihood of non-emergent ED use. Furthermore, mental health-related admissions, compared to non-mental health, will be associated with increased likelihood of emergent ED use.

**Hypothesis** **2** **(H2).** It is hypothesized that living location will moderate the impact of ED use drivers, such that for those living in a rural location (compared to an urban location) enabling resources, such as health insurance or other non-emergent sources of care, will be associated with an increased likelihood of non-emergent ED usage.

**Hypothesis** **3** **(H3).** It is hypothesized that the number of available non-emergent care sources will moderate drivers of ED usage such that, for those living in a location with zero non-emergent sources of care (compared to one or more), mental health admissions will demonstrate an increased likelihood of non-emergent ED use.

## 2. Materials and Methods

### 2.1. Study Design

Institutional Review Board approval from Old Dominion University was received prior to conducting this retrospective study. The 2014 New York State (NYS) Department of Health (DOH) Statewide Planning and Research Cooperative System (SPARCS) outpatient limited dataset, containing patient-level data for any submitting facility providing ED or ambulatory surgical services was used. All facilities in NYS providing inpatient, ambulatory surgery, ED, or outpatient services are required to submit their data to SPARCS. Variables include demographics, hospital location, patient zip code, clinical classifications software (CCS) diagnosis category, day of the week of visit, and if the visit was in the ED or not [35]. Each observation within the dataset represents a separate visit and was considered independent. Previous analyses using this data exist in the literature [36,37,38,39,40]; however, none of the prior studies have comprehensively examined correlates of non-emergent ED use from a conceptual framework nor living location or non-emergent care source availability as moderating factors. 

### 2.2. Setting

The study population consisted of all ED visits within the 2014 NYS DOH SPARCS outpatient limited dataset (approximately 6.3 million observations). Observations with a blank ED Indicator variable and a Claim Type not equal to ‘E’ were excluded since they were not ED visits. Observations with a patient zip code outside NYS were also excluded. Lastly, observations where the patient age was greater than 100 years old, sex = ’unknown’, or ethnicity = ’unknown’ were excluded. 

### 2.3. Methods of Measurement (Predictor Variable Categories with Low Cell Counts (i.e., n < 10) Were Recoded as Needed)

The dependent variable of emergent status was measured as 0 = non-emergent and 1 = emergent. Non-emergent ED use was defined as a visit within the dataset occurring in the ED for Emergency Medical Treatment and Labor Act (EMTALA) screening, urgent care, unusual circumstances, ancillary services, or other internal medicine treatment, according to the primary revenue code [35]. Emergent ED use was a visit occurring in the ED for emergent circumstances, according to the primary revenue code [35]. The constructs of the modified behavioral model comprised the independent variables for this study, as defined below. 

#### 2.3.1. Predisposing Characteristics

Age was age at the time of the visit. Race categories used for analysis included: white, African American, Asian/Pacific Islander, and other. Sex was categorized as male and female. Ethnicity used for analysis included: not of Spanish/Hispanic origin, Spanish/Hispanic origin, or Other. 

#### 2.3.2. Enabling Resources

Access to health insurance was measured using the first source of payment listed (self-pay, Medicaid, insurance company, etc.). Day of the week of the visit was categorized as either weekday or weekend using the visit day of the week. Monday through Friday was categorized as a weekday, and Saturday and Sunday was categorized as the weekend [14,15]. Time of day of the visit was categorized as either within business hours or outside of business hours using the hour recorded when the patient checked in. The hours of eight o’clock in the morning to four fifty-nine in the evening were categorized as ‘within business hours’ and five o’clock in the evening to seven fifty-nine in the morning were categorized as ‘outside of business hours’ [16,41,42].

Living location was measured as urban or rural via patient zip code and corresponding county, with a rural living location defined as any county with less than 50,000 residents, and an urban living location defined as any county with 50,000 or more residents [43]. Non-emergent care sources were measured as ‘none’ (zero non-emergent sources available), ‘single’, (one non-emergent care source available) or ‘multi’ (more than one non-emergent care source available). The number of non-emergent sources in a zip code was determined using a list of facilities in NYS maintained by the NYS DOH [44]. Facilities, such as primary care offices, medical centers, family health centers, community health centers, diagnostics and treatment centers, urgent care clinics, and 24-h pharmacy clinics, were reviewed for services offered and current operating status before being included or excluded as a non-emergent source.

#### 2.3.3. Need

Consistent with SPARCS data definitions, presenting with a chronic disease as the chief complaint was determined by the admission diagnosis equaling an ICD-9 code for conditions including, but not limited to, ALS, Alzheimer’s Disease, dementia, arthritis, asthma, cancer, chronic obstructive pulmonary disease, cystic fibrosis, diabetes, eating disorders, heart disease, obesity, osteoporosis, or reflex sympathetic dystrophy syndrome [45]. Otherwise, the ‘admission chronic disease/illness’ variable was considered as not presenting for a chronic disease/illness. All other diagnosis fields other than ‘admission diagnosis’ were then examined to better estimate if the ED visit could have been the result of disease co-morbidity. If any other diagnosis field equaled an ICD-9 code for one of the previously mentioned chronic diseases/illnesses then the ‘any chronic disease/illness’ variable was recorded as ‘presence of a chronic disease/illness’. Otherwise, the ‘any chronic disease/illness’ variable was recorded as ‘absence of a chronic disease/illness’. The ‘admission mental health’ variable equaled ‘presented due to mental health disease/illness’ if the admission diagnosis field contained an ICD-9 code for depression, anxiety, post-traumatic stress, schizophrenia, suicide, self-harm, or alcohol or substance abuse [5,21,26,46]. Otherwise the ‘admission mental health’ variable equaled ‘did not present due to mental health disease/illness’.

### 2.4. Data Analysis

Descriptive statistics were performed to assess rates of variables of interest. Logistic regression was used to test the relationship between predisposing factors, enabling resources, and need, with the criterion variable of non-emergent ED use. The categories used as reference groups (i.e., coded as 0) were chosen either because they represented the majority group or are supported by prior literature. They were: white race, male sex, non-Hispanic ethnicity, self-pay insurance, outside of business hours (time of visit), weekend (day of the week), urban living location, zero non-emergent care sources available, did not present with chronic disease (admission), no presence of chronic condition, and did not present with mental health concern. The interpretation of results was guided more by odds ratios (OR) than statistical significance due to large sample sizes and findings being used to refer to average risks in the population. Such approaches are consistent with public health approaches to big data and account for the potential of significant findings emerging by chance due to large sample size. Odds ratio interpretation was guided by recommendations in the statistical literature [47]: (1) an odds ratio up to 1.50 was considered a small association; (2) odds ratios between 1.50 and 5.00 were considered medium associations; and (3) an odds ratio of greater than 5.00 was considered a large association. Odds ratios below 1.0 were converted via calculation of the inverse (i.e., 1/OR) for ease of interpretation, with inverse odds ratios representing emergent-care findings. In accordance with statistical convention for logistic regression [48], model fit was judged using the Hosmer and Lemeshow statistic, whereas total model effect size was indicated by both Cox and Snell R^2^ and Nagelkerke R^2^ values. All analyses were performed using SPSS v22 (IBM, Armonk, NY, USA).

## 3. Results

### 3.1. Characteristics of Study Subjects

Table 1 contains predisposing characteristics, enabling resources, and need variables by type of ED use. The most notable differences between the non-emergent and emergent populations were the non-emergent population being comprised of more females, fewer minorities, more individuals not of Spanish/Hispanic origin, more individuals with a chronic disease/illness, and fewer individuals with a mental illness when compared to the emergent-use population. 

### 3.2. Main Results

#### 3.2.1. Hypothesis 1

The model included:

1. predisposing characteristics of age, gender, race, and ethnicity;
2. enabling resources of access to health insurance, day of the week of visit, time of day of visit, living location, and non-emergent care sources;
3. need variables of admission chronic disease/illness, any chronic disease/illness, and mental illness.

The overall model displayed an adequate fit to the data, Hosmer and Lemeshow Test χ^2^ (8) = 13,068.3, *p* < 0.001. Additionally, the predictors accounted for significant variance in type of ED use, χ^2^ (16) = 38,2964.5, *p* < 0.001, Cox and Snell R^2^ = 0.06, Nagelkerke R^2^ = 0.09. Table 2 contains the model statistics. Predisposing characteristics of being female (compared to males) and being of Spanish/Hispanic origin (compared to not of Spanish/Hispanic origin) were associated with an increased likelihood of non-emergent ED use (OR = 1.31 and OR = 1.28, respectively). In addition, older age appears to increase the likelihood of non-emergent ED use (OR = 1.47). The enabling resource of access to health insurance showed a small association with increased likelihood of non-emergent ED use (OR = 1.09). The need variables of having a chronic disease admission (OR = 1.58), as well as having any presence of a chronic disease (OR = 1.69), had a medium association with an increased likelihood of non-emergent ED use. 

Conversely, the following predisposing characteristics showed an increased likelihood of emergent ED use: race of African American (OR = 1.62), Asian/Pacific Islander (OR = 1.62), or other (OR = 1.34) (compared to white), and other ethnic origin (OR = 1.11) (compared to not of Spanish/Hispanic origin). The enabling resources of a visit during business hours and urban living location displayed a small association with emergent ED use (OR = 1.11). Having single or multiple non-emergent care sources (compared to none) revealed an increased likelihood of emergent ED use (OR = 1.26 and OR = 1.27, respectively). The need variable of a mental health admission indicated a medium association, yet the largest among observed predictors, with an increase in the likelihood of emergent ED use (OR = 3.51).

#### 3.2.2. Hypothesis 2

Logistic regression was again implemented to predict type of ED use within rural versus urban living locations via two separate models. Table 2 contains the model statistics for the rural-living location model, urban-living location model, and the overall model (i.e., Hypothesis 1 model) for ease of comparison.

Using only living location cases for rural-living location, the overall model displayed an adequate fit to the data, Hosmer and Lemeshow Test χ^2^ (8) = 263.6, *p* < 0.001. Additionally, the predictors accounted for significant variance in type of ED use, χ^2^ (14) = 3963.4, *p* < 0.001, Cox and Snell R^2^ = 0.02, Nagelkerke R^2^ = 0.04. Differences from the model in Hypothesis 1 included: (1) the predisposing characteristic of race no longer being a significant predictor (*p* > 0.05); (2) being of Spanish/Hispanic origin or other ethnic origin having an increased likelihood of emergent ED use (OR = 1.45 and OR = 1.29, respectively); (3) the need variable of a chronic disease admission having a moderate association with non-emergent ED use (OR = 2.12); (4) the presence of any chronic disease/illness’s association to non-emergent ED use increasing (OR = 1.78); and (5) mental illness admission’s association with emergent ED use decreasing (OR = 2.65).

Using only the observations for urban living location, the overall model displayed an adequate fit to the data, Hosmer and Lemeshow Test χ^2^ (8) = 12,869.4, *p* < 0.001. Additionally, the predictors accounted for significant variance in type of emergency department use, χ^2^ (14) = 374,903.5, *p* < 0.001, Cox and Snell R^2^ = 0.06, Nagelkerke R^2^ = 0.08. There were no meaningful changes in associations for predictors of ED use in this model compared to the overall model for Hypothesis 1. As such, only rural-living location served as a moderator for the main model effects.

#### 3.2.3. Hypothesis 3

Logistic regression was again used to predict type of ED use within zero, single, and multiple non-emergent source sub-samples via three separate models. Table 3 contains the model statistics for the non-emergent source availability models and the overall model for ease of comparison. 

Using only observations where non-emergent case sources were none, the overall model displayed adequate fit to the data, Hosmer and Lemeshow Test χ^2^ (8) = 415.3, *p* < 0.001. Additionally, the predictors accounted for significant variance in type of emergency department use, χ^2^ (14) = 23,984.6, *p* < 0.001, Cox and Snell R^2^ = 0.04, Nagelkerke R^2^ = 0.07. Differences from the model in Hypothesis 1 included: (1) a medium association between being African American or Asian/Pacific Islander and emergent ED use (OR = 1.85 and OR = 1.73, respectively) (compared to white); (2) an increased likelihood of people of Spanish/Hispanic origin using the ED for non-emergent reasons (OR = 1.66) (compared to people not of Spanish/Hispanic origin); and (3) a higher likelihood of people of other ethnic origins using the ED for emergent reasons (OR = 1.31) (compared to people not of Spanish/Hispanic origin).

Using only observations where non-emergent care sources were single, the overall model displayed adequate fit to the data, Hosmer and Lemeshow Test χ^2^ (8) = 3226.1, *p* < 0.001. Additionally, the predictors accounted for significant variance in type of emergency department use, χ^2^ (14) = 119,532.5, *p* < 0.001, Cox and Snell R^2^ = 0.06, Nagelkerke R^2^ = 0.08. The primary difference between this model and findings from the model in Hypothesis 1 was a medium association between Spanish/Hispanic origin (compared to not of Spanish/Hispanic origin) and the likelihood of non-emergent ED use (OR = 1.72). 

Using only observations where non-emergent care sources equaled multiple, the overall model displayed an adequate fit to the data, Hosmer and Lemeshow Test χ^2^ (8) = 10,729.2, *p* < 0.001. Additionally, the predictors accounted for significant variance in type of emergency department use, χ^2^ (14) = 228,607.8, *p* < 0.001, Cox and Snell R^2^ = 0.06, Nagelkerke R^2^ = 0.08. Primary differences between this model and the findings from the model in Hypothesis 1 include: (1) the predisposing factor of Spanish/Hispanic Origin (compared to not of Spanish/Hispanic origin) having a smaller association with non-emergent ED use; and (2) the enabling resource of an urban living location having a higher likelihood of emergent ED use (OR = 1.25).

## 4. Discussion

The findings for Hypothesis 1 are in concurrence with previous literature regarding demographic, chronic illness and mental illness driving ED usage [9,16,22,23]; however, our analyses offer nuance in distinguishing variables such as chronic illness and mental illness as differential risk factors for type of ED usage. Also, contrary to Anderson’s [33] supposition that enabling resources and need were the strongest healthcare utilization predictors, this study’s overall model revealed need and predisposing factors to be most influential on non-emergent ED use. In particular, the predisposing characteristics of being of older age, female, or of Spanish/Hispanic origin were associated with an increased likelihood of using the ED for non-emergent reasons [36,49]. The need variables concerning chronic disease admission also showed an increased likelihood of non-emergent ED use [10,13,21,24,25,26]. This may be due to individuals with chronic conditions generally being sicker and potentially using more medical resources [28]. With EMTALA prohibiting disposition of any patient presenting at the ED until they have been medically assessed, regardless of their ability to pay, the ED is always available for patient use [6,9,22,50,51,52,53].

A sub-category of chronic disease or illness, mental illness, has previously been shown to be associated with high rates of overall ED use [19]. However, this study revealed having a mental health condition, including suicide attempts or self-harm, as a reason for emergent ED use. This association may be a reflection of access to mental healthcare, which has been shown to be hindered by distance to a provider, geography, and provider shortages, especially in rural areas [46,54]. Mental health patients potentially not having access to a primary-care provider or mental health specialist could result in not having the necessary resources available for regular treatment and, therefore, not seeking care until it is of emergent level in order to avoid costs they cannot afford. Additionally, patients presenting with first onset and/or genuine crisis-type mental health emergencies resulting from manic episodes, psychotic breaks, and suicides or self-harm are often transported via ambulance or law enforcement to an ED for immediate treatment [55], contributing to the increase in the number of emergent mental health visits occurring in the ED.

Need was most influential in driving non-emergent ED use for Hypothesis 2, followed by enabling resources and predisposing characteristics. Race, which was previously influential in non-emergent ED use in the overall model as in other studies [7,21,23,49], was no longer a significant predictor among patients with a rural living location. All ethnic minority groups were more likely to use the ED for emergent purposes in rural areas when compared to the original model. This could be the result of cultural or social norms, such as the use of folk remedies, as a preliminary method of healthcare [56] despite the lack of empirically supported evidence [57]. Complications and increased morbidity that may result from such cultural norms can require emergent treatment to counteract the effects of the folk remedy administered [56].

Need was shown to be more influential in driving non-emergent ED use than the predisposing factors and enabling resources when zero non-emergent care sources were present. Following prior literature [22], individuals with a chronic disease/illness admission or the presence of a chronic disease/illness were more likely to use the ED for non-emergent reasons. A higher likelihood of individuals of Spanish/Hispanic origin using the ED for non-emergent reasons was also observed, possibly due to the ED being the only accessible and convenient source of healthcare. It has been previously reported that Hispanic persons are more likely to report a difficulty in finding transportation to medical care [58], conceivably resulting from low SES [59], a historic disparity for Hispanic persons when compared to non-Hispanics [60]. Additionally, individuals with a low SES who typically walk or use public transportation to get to medical care are less likely to have a regular source of care [61]. The non-emergent ED use observed for the Spanish/Hispanic population in this study may also be influenced by the type of health insurance obtained and knowledge regarding where to go for non-emergent care. Upon the Medicaid expansion and implementation of the Patient Protection and Affordable Care Act [61], some states experienced higher non-emergent ED use potentially due to newly covered individuals who did not necessarily have a regular source of care [6].

### 4.1. Implications for Service Delivery

This study revealed mental health-related ED visits in NYS, including those related to suicide and self-harm, increase the likelihood of emergent ED visits (demonstrating the largest effect on ED usage overall). Since emergency care providers have a high likelihood of dealing with mental health emergencies, it may be beneficial to provide additional mental health training and education for emergency department staff. Such training may help ensure that mental health patients receive the best treatment possible upon their arrival at the ED. The minimal education ED staff receive is likely that provided during medical education or postgraduate training [62]. Furthermore, ED staff often state that they do not feel adequately educated in assessing and diagnosing mental health diseases; therefore, increasing mental health training concerning clinical assessment and the immediate management of mental health patients in the ED during medical education or postgraduate education may help to decrease any feelings of inadequacy [62,63]. This could be done through more coursework or rotations through both the ED and mental health, in order for medical staff to gain more familiarity with what they will likely encounter during practice.

Training for non-mental health healthcare providers has previously been available to help with learning to assess potential at-risk mentally ill populations for conditions such as suicide (i.e., ‘gatekeeper training’) [64,65]. Such training has been seen to assist in improving the knowledge, attitudes and skills of individuals likely to encounter at-risk populations [65] by focusing on learning how to recognize the signs and symptoms of psychological distress, improving communication with at-risk patients, understanding how to manage risk if suicide is a concern, understanding where to refer or bring at-risk patients, and knowing how to refer at-risk patients to specified resources [64]. Scholars have even called for training and empirical testing on the applicability of gatekeeper training for overall mental illness so that ED staff can recognize these encounters [66]. As ED providers become more educated on the best way to treat the patient population, ideas for best practices will become the norm. The ideas and methods discovered by ED staff will likely be of value for various levels of policymakers as they evaluate current emergency and mental healthcare policies, or as new policies undergo development that would better serve the patient population. Additionally, as many hospitals contain consultation-liaison services, integrated training with this service for all ED trainees may also be of value for providing healthcare to the patients with mental health concerns seen in the ED.

Historically, medical consultations in the ED from mental health providers are not appropriately used. Training on when to request a mental health consultation during initial medical education or postgraduate education may also assist in standardizing the use of mental health resources in an ED. Having a mental health provider on staff in the ED may also help with better regulating the use of mental health resources [63]. This would be consistent with recommendations in the literature suggesting that mental health providers move towards new settings and non-traditional career paths [67].

### 4.2. Limitations

One limitation of this study was that all variables except day of the week of visit varied significantly by type of ED use; however, large sample sizes likely account for some of the significant findings. Multiple observations from the same patient were also not accounted for, as this was a visit-level analysis. The capacity to manage the number of patients presenting to non-emergent sources at any given time was not accounted for in this study, as this analysis focused primarily on the total number of non-emergent sources potentially available in a zip code. Additionally, this study was retrospective and examined one year of data, preventing any inference of causal relationships. The study sample also only included those from NYS, which varies demographically from other locations in the U.S., and did not address all racial/ethnic groups, possibly limiting generalizability. Future studies should analyze non-emergent ED use over time in order to explore the possibility of causal relationships, and ED use on public holidays when an individual’s mental health may be more greatly impacted and other sources of care may be less available. Further work can expand study samples to include ED visits from other states, break out the summary variable of chronic disease and mental health to explore the individual impact of each disease, and include all racial/ethnic groups to help increase generalizability.

## 5. Conclusions

Further educating ED staff through gatekeeper training may aid in driving changes in how healthcare, especially mental healthcare, is delivered. Currently, the ED serves as a healthcare resource that is available 24 h. The ED will most likely continue to be a non-emergent care ‘safety net’, especially for those without a primary-care provider, unless programs and clinics are developed for non-emergent care. However, these changes require new payment incentives and disease-management strategies to assist in making healthcare more accessible. Improving access to healthcare, including care for mental health conditions such as attempted suicide and self-harm, may be achieved by applying the findings of this study, educating ED staff to better manage the mental healthcare needs of the patients they see, and changing systems for mental healthcare delivery in order to expand care options for patients.

## Figures and Tables

**Table 1:** Predisposing characteristic, enabling resource, and need comparisons by type of emergency department use.

Model Variable	Total Sample (*n* = 6,291,158)	Non-Emergent (*n* = 4,693,638)	Emergent (*n* = 1,597,520)	Test-Statistic
**Predisposing Characteristics**				
Age (st dev)	36.3 (22.7)	38.8 (22.5)	29.1 (22.1)	t (6,291,156) = 473.1 *
Sex				χ^2^ [1] = 24530.3 *
Male	2,849,958 (45.3%)	2,041,150 (43.5%)	808,808 (50.6%)	
Female	3,441,200 (54.7%)	2,652,488 (56.5%)	788,712 (49.4%)	
Race				χ^2^ [3] = 91836.6 *
White	2,895,537 (46.0%)	2,324,034 (49.5%)	571,503 (35.7%)	
African American	1,700,966 (27.0%)	1,180,106 (25.1%)	520,860 (32.6%)	
Asian/Pacific Islander	167,168 (2.6%)	113,381 (2.4%)	53,787 (3.4%)	
Other	1,513,468 (24.0%)	1,064,956 (22.7%)	448,512 (28.1%)	
Ethnicity				χ^2^ [2] = 16862.6 *
Not Spanish/Hispanic	5,001,770 (79.5%)	3,785,538 (80.6%)	1,216,232 (76.1%)	
Spanish/Hispanic	53,212 (0.8%)	41,783 (0.9%)	11,429 (0.7%)	
Other	1,236,176 (19.6%)	866,317 (18.4%)	369,859 (23.1%)	
**Enabling Resources**				
Access to Health Insurance				χ^2^ [1] = 6584.9 *
Yes	5,628,419 (89.5%)	4,226,386 (90.0%)	1,402,033 (87.7%)	
No	662,739 (10.5%)	467,252 (10.0%)	195,487 (12.2%)	
Day of the Week of Visit				χ^2^ [1] = 0.02
Weekday	4,574,489 (72.7%)	3,412,810 (72.7%)	1,161,679 (72.7%)	
Weekend	1,716,669 (27.3%)	1,280,828 (27.3%)	435,841 (27.3%)	
Time of Day of Visit				χ^2^ [1] = 436.2 *
Within Business Hours	3,365,728 (53.5%)	2,499,693 (53.2%)	866,035 (54.2%)	
Outside Business Hours	2,925,430 (46.5%)	2,193,945 (46.8%)	731,485 (45.7%)	
Living Location				χ^2^ [1] = 4779.9 *
Urban	6,128,318 (97.4%)	4,560,163 (97.1%)	1,568,155 (98.2%)	
Rural	162,840 (2.6%)	133,475 (2.8%)	29,365 (1.8%)	
Non-Emergent Care Sources				χ^2^ [2] = 12147.2 *
None	533,403 (8.5%)	431,355 (9.2%)	102,048 (6.4%)	
Single	2,059,493 (32.7%)	1,529,206 (32.6%)	530,287 (33.2%)	
Multiple	3,698,262 (58.8%)	2,733,077 (58.2%)	965,185 (60.4%)	
**Need**				
Chronic Disease Admission				χ^2^ [1] = 330.4 *
Yes	388,489 (6.2%)	294,616 (6.3%)	93,873 (5.8%)	
No	5,902,669 (93.8%)	4,399,022 (93.7%)	1,503,647 (94.1%)	
Any Chronic Disease				χ^2^ [1] = 90409.4 *
Yes	1,840,259 (29.2%)	1,522,292 (32.4%)	317,967 (20.0%)	
No	4,450,899 (70.7%)	3,171,346 (67.6%)	1,279,553 (80.0%)	
Mental Health Admission				χ^2^ [1] = 6227.1 *
Yes	225,504 (3.6%)	152,226 (3.2%)	73,278 (4.6%)	
No	6,065,654(96.4%)	4,541,412 (96.7%)	1,524,242 (95.4%)	

Notes: * *p* < 0.001.

**Table 2:** Overall emergency department use compared to rural and urban living location emergency department use.

Model Variables	Overall				Rural Living Location				Urban Living Location			
	B (SE)	Wald χ^2^ (df)	*p*	OR (95% CI)	B (SE)	Wald χ^2^ (df)	*p*	OR (95% CI)	B (SE)	Wald χ^2^ (df)	*p*	OR (95% CI)
Intercept	−1.47 (0.008)	33,438.7 (1)	<0.001	0.23	−1.41 (0.031)	2073.3 (1)	<0.001	0.24	−1.31 (0.005)	66,112.7 (1)	<0.001	0.27
Age	−0.38 (0.001)	129,323.5 (1)	<0.001	0.68 (0.68–0.68)	−0.34 (0.007)	2330.5 (1)	<0.001	0.71 (0.71–0.72)	−0.38 (0.001)	19,689.1 (1)	<0.001	0.68 (0.68–0.68)
Female	−0.27 (0.002)	19,757.4 (1)	<0.001	0.76 (0.76–0.77)	−0.16 (0.013)	145.8 (1)	<0.001	0.85 (0.83–0.87)	−0.27 (0.002)	19,689.1 (1)	<0.001	0.76 (0.76–0.77)
White (ref)	-	46,081.4 (3)	<0.001	-	-	**0.78 (3)**	**0.85**	-	-	46,081.1 (3)	<0.001	-
African American	0.48 (0.002)	43,016.5 (1)	<0.001	1.62 (1.61–1.63)	**0.03 (0.040)**	**0.65 (1)**	**0.42**	**1.03 (0.95–1.12)**	0.48 (0.002)	43,056.4 (1)	<0.001	1.62 (1.62–1.63)
Asian/Pacific Islander	0.48 (0.006)	7477.9 (1)	<0.001	1.62 (1.61–1.64)	**−0.04 (0.139)**	**0.09 (1)**	**0.75**	**0.96 (0.73–1.26)**	0.49 (0.006)	7486.1 (1)	<0.001	1.63 (1.61–1.64)
Other Race	0.29 (0.003)	10,711.7 (1)	<0.001	1.34 (1.33–1.34)	**−0.01 (0.040)**	**0.02 (1)**	**0.87**	**0.99 (0.92–1.07)**	0.29 (0.003)	10,737.7 (1)	<0.001	1.34 (1.33–1.34)
Not Spanish/Hispanic (ref)	-	2122.3 (2)	<0.001	-	-	38.6 (2)	<0.001	-	-	2110.2 (2)	<0.001	-
Spanish/Hispanic	−0.25 (0.011)	522.7 (1)	<0.001	0.78 (0.76–0.79)	**0.37 (0.093)**	**15.8 (1)**	**<0.001**	**1.45 (1.21–1.74)**	−0.26 (0.011)	539.7 (1)	<0.001	0.77 (0.76–0.79)
Other Origin	0.10 (0.003)	1380.5 (1)	<0.001	1.11 (1.10–1.11)	**0.25 (0.045)**	**31.7 (1)**	**<0.001**	**1.29 (1.18–1.41)**	0.10 (0.003)	1351.8 (1)	<0.001	1.11 (1.10–1.11)
Health Insurance Access	−0.09 (0.003)	1021.0 (1)	<0.001	0.91 (0.90–0.92)	−0.15 (0.025)	35.2 (1)	<0.001	0.86 (0.82–0.90)	−0.09 (0.003)	982.4 (1)	<0.001	0.91 (0.90–0.92)
Weekday Visit	0.01 (0.002)	7.4 (1)	<0.001	1.01 (1.00–1.01)	−0.06 (0.014)	17.8 (1)	<0.001	0.94 (0.91–0.97)	0.01 (0.002)	11.3 (1)	<0.001	1.01 (1.00–1.01)
Within Business Hours	0.10 (0.002)	3047.2 (1)	<0.001	1.11 (1.11–1.11)	0.08 (0.013)	39.8 (1)	<0.001	1.09 (1.06–1.11)	0.11 (0.002)	3008.1 (1)	<0.001	1.11 (1.11–1.12)
Urban Living Location	0.16 (0.007)	576.1 (1)	<0.001	1.17 (1.16–1.19)	-	-	-	-	-	-	-	-
No Non-Emergent Sources (ref)	-	4040.8 (2)	<0.001	-	-	150.1 (2)	<0.001	-	-	3920.4 (2)	<0.001	-
Single Non-Emergent Source	0.23 (0.004)	3329.7 (1)	<0.001	1.26 (1.25–1.27)	0.22 (0.018)	148.5 (1)	<0.001	1.23 (1.20–1.29)	0.23 (0.004)	3195.4 (1)	<0.001	1.26 (1.25–1.27)
Multiple Non-Emergent Source	0.24 (0.004)	4001.5 (1)	<0.001	1.27 (1.26–1.28)	0.16 (0.018)	82.2 (1)	<0.001	1.18 (1.14–1.22)	0.24 (0.004)	3896.0 (1)	<0.001	1.28 (1.27–1.28)
Chronic Disease Admission	−0.46 (0.008)	3460.3 (1)	<0.001	0.63 (0.62–0.64)	−0.75 (0.070)	115.6 (1)	<0.001	0.47 (0.41–0.54)	−0.46 (0.008)	3316.6 (1)	<0.001	0.63 (0.62–0.64)
Any Chronic Disease	−0.53 (0.003)	41,898.3 (1)	<0.001	0.59 (0.58–0.59)	−0.17 (0.016)	109.3 (1)	<0.001	0.84 (0.82–0.87)	−0.53 (0.003)	42,160.9 (1)	<0.001	0.58 (0.58–0.59)
Mental Health Admission	1.26 (0.009)	20,006.5 (1)	<0.001	3.51 (3.45–3.57)	0.97 (0.081)	145.7 (1)	<0.001	2.65 (2.26–3.10)	1.26 (0.009)	19,897.0 (1)	<0.001	3.53 (3.46–3.59)

Notes: SE = Standard error; df = Degrees of freedom; OR = Odds ratio; CI = Confidence interval; ref = Reference group; Bold font denotes notable changes in a sub-group model compared to the main model.

**Table 3:** Overall emergency department use compared to no non-emergent care sources emergency department use.

Model Variables	Overall				No Non-Emergent Care Sources				Single Non-Emergent Care Source				Multiple Non-Emergent Care Sources			
	B (SE)	Wald χ^2^ (df)	*p*	OR (95% CI)	B (SE)	Wald χ^2^ (df)	*p*	OR (95% CI)	B (SE)	Wald χ^2^ (df)	*p*	OR (95% CI)	B (SE)	Wald χ^2^ (df)	*p*	OR (95% CI)
Intercept	−1.47 (0.008)	33,438.7 (1)	<0.001	0.23	−1.52 (0.020)	5624.0 (1)	<0.001	0.22	−1.21 (0.012)	9778.1 (1)	<0.001	0.29	−1.26 (0.011)	13,110.8 (1)	0	0.28
Age	−0.38 (0.001)	129,323.5 (1)	<0.001	0.68 (0.68–0.68)	−0.35 (0.004)	8019.4 (1)	<0.001	0.70 (0.69–0.71)	−0.35 (0.002)	36,382.6 (1)	<0.001	0.70 (0.70–0.71)	−0.40 (0.001)	85,494.7 (1)	<0.001	0.67 (0.67–0.67)
Female	−0.27 (0.002)	19,757.4 (1)	<0.001	0.76 (0.76–0.77)	−0.21 (0.007)	873.4 (1)	<0.001	0.81 (0.79–0.82)	−0.27 (0.003)	6726.7	<0.001	0.76 (0.76–0.77)	−0.27 (0.002)	12,151.7 (1)	<0.001	0.76 (0.76–0.76)
White (ref)	-	46,081.4 (3)	<0.001	-	-	4787.6 (3)	<0.001	-	-	15,939.9 (3)	<0.001	-	-	25,670.3 (3)	<0.001	-
African American	0.48 (0.002)	43016.5 (1)	<0.001	1.62 (1.61–1.63)	**0.62 (0.009)**	**4516.3 (1)**	**<0.001**	**1.85 (1.82–1.89)**	0.48 (0.004)	15,106.0 (1)	<0.001	1.62 (1.61–1.64)	0.47 (0.003)	23,627.6 (1)	<0.001	1.59 (1.59–1.61)
Asian/Pacific Islander	0.48 (0.006)	7477.9 (1)	<0.001	1.62 (1.61–1.64)	**0.55 (0.024)**	**531.0 (1)**	**<0.001**	**1.73 (1.65–1.82)**	0.48 (0.010)	2133.8 (1)	<0.001	1.62 (1.59–1.66)	0.47 (0.007)	4751.4 (1)	<0.001	1.61 (1.59–1.63)
Other Race	0.29 (0.003)	10,711.7 (1)	<0.001	1.34 (1.33–1.34)	**0.16 (0.012)**	**169.4 (1)**	**<0.001**	**1.17 (1.15–1.20)**	0.29 (0.000)	3559.9 (1)	<0.001	1.35 (1.33–1.36)	0.29 (0.004)	6828.7 (1)	<0.001	1.34 (1.33–1.35)
Not Spanish/Hispanic (ref)	-	2122.3 (2)	<0.001	-	-	426.2 (2)	<0.001	-	-	1134.1 (2)	<0.001	-	-	942.3 (2)	<0.001	-
Spanish/Hispanic Origin	−0.25 (0.011)	522.7 (1)	<0.001	0.78 (0.76–0.79)	**−0.51 (0.061)**	**69.9 (1)**	**<0.001**	**0.60 (0.53–0.67)**	**−0.54 (0.020)**	**746.9 (1)**	**<0.001**	**0.58 (0.56–0.61)**	−0.08 (0.014)	33.2 (1)	<0.001	0.92 (0.90–0.95)
Other Origin	0.10 (0.003)	1380.5 (1)	<0.001	1.11 (1.10–1.11)	**0.27 (0.015)**	**333.6 (1)**	**<0.001**	**1.31 (1.27–1.35)**	0.08 (0.005)	264.7 (1)	<0.001	1.08 (1.07–1.09)	0.10 (0.003)	859.9 (1)	<0.001	1.11 (1.10–1.11)
Health Insurance Access	−0.09 (0.003)	1021.0 (1)	<0.001	0.91 (0.90–0.92)	−0.02 (0.012)	3.5 (1)	0.06	0.98 (0.95–1.00)	−0.07 (0.005)	230.6 (1)	<0.001	0.93 (0.92–0.94)	−0.11 (0.004)	861.7 (1)	<0.001	0.89 (0.88–0.90)
Weekday Visit	0.01 (0.002)	7.4 (1)	<0.001	1.01 (1.00–1.01)	−0.00 (0.008)	0.40 (1)	0.53	0.99 (0.98–1.01)	0.01 (0.004)	3.5 (1)	<0.001	1.01 (1.00–1.01)	0.01 (0.003)	5,7 (1)	0.02	1.01 (1.00–1.01)
Within Business Hours	0.10 (0.002)	3047.2 (1)	<0.001	1.11 (1.11–1.11)	0.11 (0.007)	230.6 (1)	<0.001	1.11 (1.10–1.13)	0.11 (0.003)	1213.8 (1)	<0.001	1.12 (1.11–1.16)	0.09 (0.002)	1622.0 (1)	<0.001	1.10 (1.10–1.11)
Urban Living Location	0.16 (0.007)	576.1 (1)	<0.001	1.17 (1.16–1.19)	0.09 (0.016)	38.2 (1)	<0.001	1.10 (1.07–1.13)	0.13 (0.011)	130.4 (1)	<0.001	1.13 (1.11–1.16)	0.22 (0.010)	463.3 (1)	<0.001	1.25 (1.22–1.27)
No Non-Emergent Sources (ref)	-	4040.8 (2)	<0.001	-	-	-	-	-	-	-	-	-	-	-	-	-
Single Non-Emergent Source	0.23 (0.004)	3329.7 (1)	<0.001	1.26 (1.25–1.27)	-	-	-	-	-	-	-	-	-	-	-	-
Multiple Non-Emergent Source	0.24 (0.004)	4001.5 (1)	<0.001	1.27 (1.26–1.28)	-	-	-	-	-	-	-	-	-	-	-	-
Chronic Disease Admission	−0.46 (0.008)	3460.3 (1)	<0.001	0.63 (0.62–0.64)	−0.47 (0.034)	191.7 (1)	<0.001	0.62 (0.58–0.67)	−0.44 (0.013)	1109.9 (1)	<0.001	0.64 (0.63–0.66)	−0.47 (0.010)	2141.1 (1)	<0.001	0.62 (0.61–0.63)
Any Chronic Disease	−0.53 (0.003)	41,898.3 (1)	<0.001	0.59 (0.58–0.59)	−0.48 (0.009)	2717.5 (1)	<0.001	0.62 (0.61–0.63)	−0.57 (0.004)	16,564.2 (1)	<0.001	0.56 (0.56–0.57)	−0.51 (0.003)	22,697.9 (1)	<0.001	0.60 (0.59–0.61)
Mental Health Admission	1.26 (0.009)	20,006.5 (1)	<0.001	3.51 (3.45–3.57)	1.23 (0.038)	1035.6 (1)	<0.001	3.42 (3.17–3.69)	1.34 (0.015)	8276.2 (1)	<0.001	3.84 (3.73–3.95)	1.20 (0.012)	10,694.3 (1)	<0.001	3.23 (3.25–3.39)

Notes: SE = Standard error; df = Degrees of freedom; OR = Odds ratio; CI = Confidence interval; ref = Reference group; Bold font denotes notable changes in each sub-group model compared to the main model.
